# A randomised controlled feasibility trial to evaluate local heat preconditioning on wound healing after reconstructive breast surgery: the preHEAT trial

**DOI:** 10.1186/s40814-019-0392-y

**Published:** 2019-01-11

**Authors:** Saahil Mehta, Suzie Cro Cro, Billie Coomber, Rachel Rolph, Victoria Cornelius, Jian Farhadi

**Affiliations:** 1 Department of Plastic Surgery, Guy’s and St. Thomas’ NHS Foundation Trust, Westminster Bridge Road, London, SE1 7EH UK; 2 Imperial College Trials Unit, 1st Floor, Stadium House, 68 Wood Lane, London, W12 7RH UK; 3 Department of Plastic Surgery, Faculty of Medicine, University of Basel, Klingelbergstrasse 61, CH - 4046 Basel, Switzerland

## Abstract

**Objective:**

preHEAT was a randomised controlled feasibility trial to determine how best to measure skin necrosis in breast reconstruction to inform the design of a larger multicentre trial.

**Background:**

Mastectomy skin flap necrosis (MSFN) is a serious complication resulting in prolonged wound healing. Local heat preconditioning of the MSF before surgery has been shown to reduce skin necrosis in immediate breast reconstruction patients (IBR).

**Method:**

preHEAT was a single-centre, randomised control two-arm single-blind parallel arm feasibility trial of local heat preconditioning in breast cancer patients undergoing SSM and NSM at Guy’s and St Thomas’ Hospital, London, UK. All patients undergoing IBR above the age of 18 were included. Intervention patients heated breast skin to 43 °C in three, 30-min cycles interrupted by spontaneous cooling using hot water bottles. The primary aim was to compare measurement of skin necrosis using binary ‘yes/no’ assessment, the SKIN score, and wound area.

**Results:**

One hundred forty-one patients were randomised over a 2-year period (71 heated group, 70 controls). There was near perfect agreement between assessors using the “yes/no” measurement of necrosis. The proportion of patients experiencing necrosis in controls was 35% (*n* = 23/66) in the heated 26% (*n* = 18/68]). In the control group, 17% (*n* = 4/23) patients experiencing necrosis required surgical intervention for necrosis compared to 11% (*n* = 2/18) in the heated group.

**Conclusion:**

The binary outcome of MSFN “yes/no” is a suitable and reliable primary outcome measure of necrosis and was superior to the SKIN Score or necrosis area. The trial study design is feasible for a larger definitive trial.

**Trial registration:**

ISRCTN15744669. Date of registration: 25/02/2018

**Electronic supplementary material:**

The online version of this article (10.1186/s40814-019-0392-y) contains supplementary material, which is available to authorized users.

## Background

According to the most recent NICE guidance, immediate breast reconstruction should be available to all women requiring a mastectomy for breast cancer in the UK [1]. Skin sparing mastectomy (SSM) and nipple sparing mastectomy (NSM) are becoming more commonly performed and allow for cosmetically superior results. In 2011, 16,485 women underwent mastectomy in the UK [2]. However, due to the delicate blood supply to the skin of the mastectomy skin flap, it is often susceptible to mastectomy skin flap necrosis (MSFN). This can require further surgical interventions, delayed recovery and an increased length of stay (LOS) in hospital, which can cause a delay in the delivery of adjuvant cancer treatment and compromise oncological outcomes. SSM and NSM with reconstruction are already costly procedures hence reducing the incidence of skin necrosis to improve patient recovery and to reduce the financial burden to the NHS is of high interest.

Experimental results have shown that applying heat to the skin before surgery can reduce the incidence of skin necrosis by improving the blood supply in animal models [3–6]. The proposed mechanism is via the induction of heat shock proteins, specifically HSP-32 and a local release of carbon monoxide, a potent vasodllator. The animals in these experiments received pulsatile heat to an area of their skin before a small pedicled skin-flap was raised in a dorsal skin chamber. These studies were not specifically using breast skin but nevertheless provided mechanistic evidence for local heat preconditioning that could be applied in the clinical situation. In a small translational pilot study in our own department, we used hot water bottles to simulate the pulsatile heat described by Harder et al. to heat precondition the breast skin envelope preoperatively. We observed a 26% absolute reduction (95% CI [10% to 36%]) of skin necrosis in high-risk patients undergoing SSM and reconstruction [7].

Local heat preconditioning has the potential to be a safe and cost-effective method of reducing skin necrosis that could be implemented easily as part of everyday practice in the NHS. preHEAT was a feasibility study for a trial that would evaluate local heat preconditioning with respect to its effects on skin necrosis in patients with breast cancer undergoing SSM and NSM. The overarching aim of preHEAT was to create a robust protocol that can be used for a larger definitive multicentre trial. The primary objective of the study was to identify the best way to measure mastectomy skin flap necrosis and to estimate the necrosis event rate in each treatment group using this definition to inform the design of a definitive trial in breast cancer patients undergoing SSM and NSM. Secondary feasibility objectives included estimating the recruitment rate and 30–40 day follow-up; to assess adherence with the heating protocol; to estimate the treatment effect on length of hospital stay and to estimate the rates of surgical versus conservative management of skin necrosis. Further details of the trial can be found in the protocol [8] .

## Methods

preHEAT was a single centre, randomised controlled parallel two-arm single-blind feasibility trial of local heat preconditioning in breast cancer patients undergoing SSM and NSM at Guy’s and St Thomas’ Hospital NHS Foundation Trust, London, UK. The comparator intervention was mastectomy and reconstruction without any heat preconditioning**.** Ethical approval for the study in breast cancer patients undergoing SSM/NSM was given by the Health Research Authority NRES Committee South Central – Hampshire B (ethics number 14/SC/1334). During the study period, 10/2014–10/2017, we aimed to recruit 180 patients to the trial over a 24-month period.

All referrals to Plastic Surgery were screened and patients referred for mastectomy and immediate breast reconstruction were identified. These patients were approached to participate in the study during a weekly breast reconstruction clinic. The inclusion criteria were women over the age of 18 (no maximum age) undergoing SSM or NSM and immediate breast reconstruction (IBR) (autologous & implant). Exclusion criteria included: patients undergoing delayed (2-stage) reconstruction, patients with a latex allergy and patients with inflammatory breast cancer. Consenting participants were allocated to treatment group via an online randomisation system hosted by the King’s College London Clinical Trials Unit (KCTU). Allocation was undertaken (1:1) using minimisation with a 10% random component stratifying for:

- Type of reconstruction (Implant vs Autologous)
- Smoking status (Yes/No)
- Diabetic (Yes/No)
- BRCA carrier status (Yes/No)

The online data and management system (MACRO by InferMed (www.infermed.com) hosted by KCTU was used for data collection.

All SSM’s and NSM’s were performed by 8 breast surgeons and all breast reconstructions were performed by 7 plastic surgeons.

### Heating procedure

Patients allocated to the treatment group were given clear written instructions and also an in depth face-to-face explanation on how to perform the local heat preconditioning procedure. The patients were given a standard commercially available hot water bottle and a simple, accurate water thermometer. They were instructed to heat water in a saucepan to 43 °C and pour this into the hot water bottle. The water bottle was placed on the naked breast for 30 min and the skin was then allowed to cool spontaneously for 30 min. This was repeated twice with freshly heated water. For patients undergoing a unilateral operation, only that breast was heated; for bilateral operations, only the right breast was heated. Patients were asked to perform the heating procedure as close to 12 h before surgery at home. Patients attended for surgery as normal the following day.

All patients in the treatment group were given a compliance form and were asked to record both the exact temperature of the water and the times of application (including duration). The compliance forms are available as supplementary material as Additional file 4.

### Primary outcome measure

Necrosis was measured as follows:

- Necrosis present “Yes/No” (any depth): by clinical judgement
- Necrosis depth (using the SKIN score as described below): recorded independently by a primary clinical assessor and a secondary clinical assessor and from photographs by two further independent assessors.
- Necrosis area (mm^2^): recorded by area using a transparent grid independently by both a primary clinical assessor and a secondary clinical assessor.

The SKIN score classifies the depth of necrosis from A to D where: A—none, B—colour change of flap suggesting impaired perfusion or ischaemic injury, C—partial thickness skin flap necrosis resulting in breakdown of the wound and D—full thickness skin flap necrosis [9].

Necrosis assessments were made on an ongoing basis from day of first post-operative outpatient appointment (∼day 12–16), with follow-up occurring until 30–40 days post-surgery.

### Secondary outcome measures

The secondary outcome measures were as follows:

- Recruitment rate (number randomised/number eligible)
- 30–40 day follow-up rate
- Level of compliance with heating protocol
- Length of hospital stay
- Rates of surgical/conservative management of skin necrosis

### Blinding

Postoperative necrosis assessments were performed by nurses and doctors working in the Plastic Surgery Department. All outcome assessors and study team members were blinded except for the Trial Manager (BC).

Due to the nature of the intervention, participants were not blinded and were given clear instructions not discuss the trial with outcome assessors or healthcare professional staff. To maintain blinding of the double necrosis outcome assessments for each patient, the first assessor saw the patient alone, performed their assessment and placed their documented findings in a sealed envelope. This was repeated by the second assessor at a different time from the first assessor. At no point did the assessors discuss their assessments with each other or with patients.

In addition, clinical photographs were taken of all patients who developed skin necrosis with their consent. These photos were collated at the end of the trial and given to two independent clinical assessors to verify the SKIN score. These assessors had not been involved previously with the trial.

### Sample size calculation

Since the main aim of this study was to identify a suitable way to measure necrosis and to estimate the event rate in each arm, in addition to establishing recruitment rates, the sample size calculation was precision based, given the anticipated number of potentially eligible cases. Within Guy’s and St Thomas’ Hospital over 150 SSMs are performed per year. Over a 2-year recruitment period, we anticipated a total of around 300 potentially eligible individuals. This would allow us to estimate the 95% confidence interval (CI) for the recruitment rate with precision of at least ± 6 percentage points (calculation based on proportion requiring the largest sample size e.g. 50%). Based on our previous experience, we anticipated that the actual recruitment rate would be no less than 60%, so this would provide us with at least 180 participants (60% of 300) [7]. Assuming the proportion of necrosis events amongst participants in the control group was 30% a sample size of 180 would allow us to estimate ‘necrosis’ rates within each group to at least ± 9 percentage points. If the true difference between event rates were 15%, we would be able to estimate this within ± 12 percentage points. The estimated events rates in preHEAT will be used to calculate the required sample size for a larger adequately powered definitive study.

### Statistical analysis

Analysis was conducted in accordance with the preHEAT statistical analysis plan (SAP v1.0), which was finalised on June 1, 2017, prior to any unblinded data extraction. As preHEAT was a feasibility study, the analysis for this trial was primarily descriptive. Analysis followed the intention-to-treat (ITT) principle, i.e. all participants with a recorded outcome were included in the analysis according to the treatment group to which they were randomised regardless of treatment actually received. Please see Cro et al. for full details of the statistical analysis plan [8].

The agreement between the primary and secondary clinical assessments and photographic assessments of necrosis presence (Yes/No) and depth was assessed using Fleiss’ Kappa statistic [10].

The agreement between the first and second clinical assessor’s necrosis area measures at the first occasion was assessed using the method of Bland and Altman [11].

A performance matrix was constructed to assess each necrosis measurement method against three pre-specified criteria:

1. Subjectivity of measurement (using Kappa statistic)
2. Sample size required for a definitive trial to demonstrate a statistically significant difference between treatment and control groups
3. Proportion of patients with observed response

## Results

**Fig. 1:**
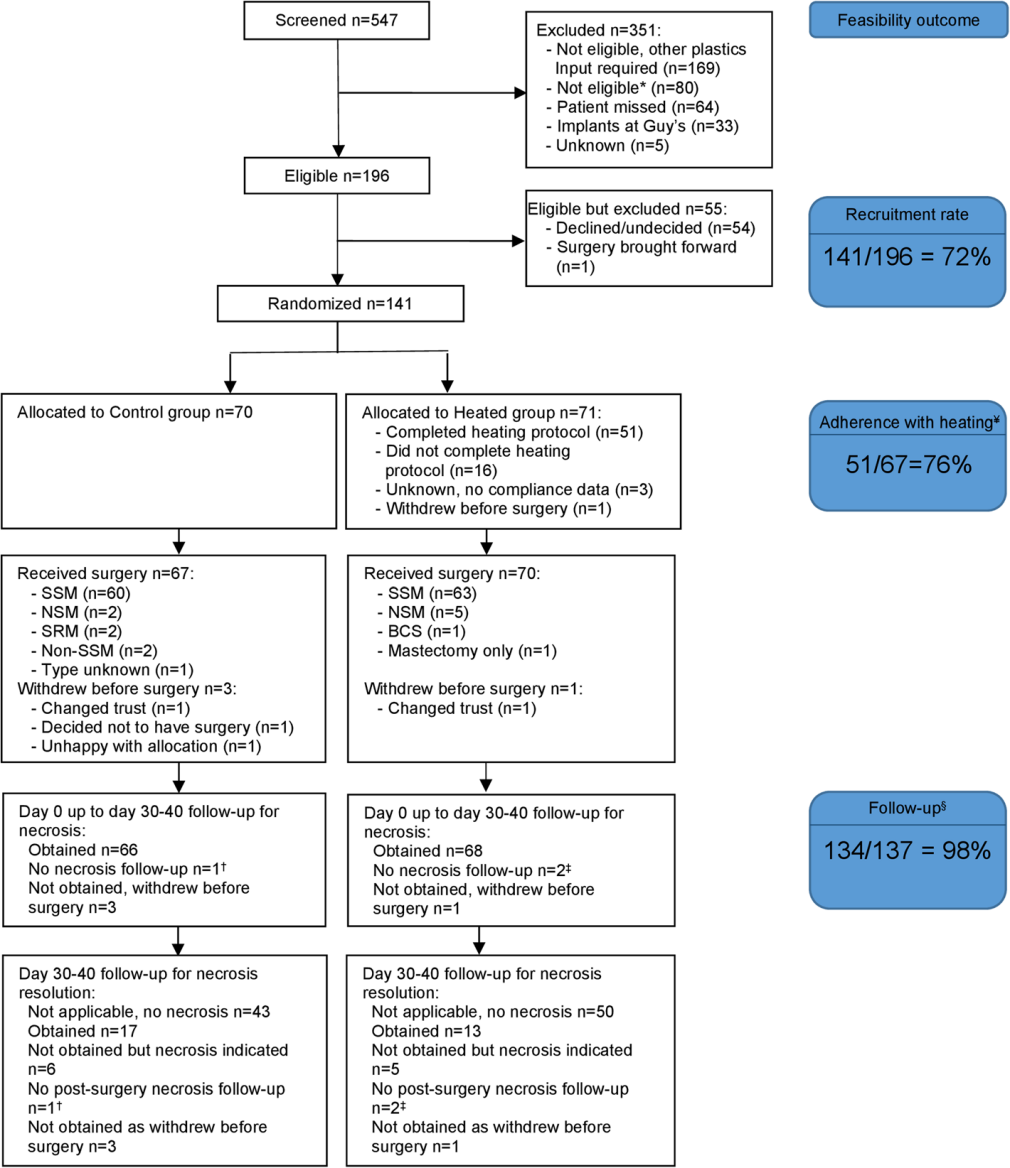
CONSORT flow diagram for the preHEAT trial showing feasibility outcome measures of recruitment rate, adherence with heating protocol and follow-up rate

Figure 1 presents the CONSORT flow diagram for this trial. Between March 9, 2015, to March 7, 2017 (the planned 2-year recruitment period). Seventy patients were allocated to the control group and 71 to the heated group. Our overall recruitment rate for the trial was 72% (141/196).

The baseline characteristics of our patient group are shown in Table 1. The mean age of patients was 50.6 years (standard deviation; SD = 9.4) with a mean body mass index of 28.1 kg/m^2^ (SD = 5.2). A total of 26 (18%) implant only based reconstructions compared with 115 (82%) autologous reconstructions were planned.

**Table 1:** Baseline and operative characteristics

	Control *n* = 70	Heated *n* = 71	Total *n* = 141
Age (mean)	50.5	50.6	50.6
BMI (kg/m^2^ mean)	28.6	27.7	28.1
Ethnicity			
White	47 (67%)	52 (73%)	99 (70%)
Black	19 (27%)	13 (18%)	32 (23%)
Mixed race	2 (3%)	3 (4%)	5 (4%)
Other	1 (1%)	2 (3%)	3 (2%)
Diabetic (*n*, %)	1 (1%)	4 (6%)	5 (4%)
Not diabetic	69 (99%)	67 (94%)	136 (96%)
Smoker (*n*, %)	8 (11%)	10 (14%)	18 (13%)
Non-smoker	62 (89%)	61 (86%)	123 (87%)
BRCA status (*n*, %)			
Carrier	13 (19%)	13% (18%)	26 (18%)
Non-carrier	57 (81%)	58 (82%)	115 (82%)
Neo-adjuvant therapy			
None	48 (69%)	53 (76%)	101 (72%)
Chemotherapy only	16 (23%)	11 (16%)	27 (19%)
Radiotherapy only	2 (3%)	2 (3%)	4 (3%)
Chemotherapy and radiotherapy	4 (6%)	4 (6%)	8 (6%)
Preoperative details			
Sternal notch to nipple distance (cm, mean)	25.6 (*n* = 47)	25.3 (*n* = 48)	25.5
Breast cup size	*n* = 64	*n* = 66	
A-D	43	43	–
DD-JJ	21	23	–
Operative details			
Type of reconstruction			
Implant	13 (19%)	13 (18%)	26 (18%)
Autologous	57 (81%)	58 (82%)	115 (82%)
Type of mastectomy	*n* = 66	*n* = 70	–
SSM	60 (91%)	63 (93%)	–
NSM	2 (3%)	5 (7%)	–
Other	4 (6%)	2 (2%)	–

Four (3%) randomised patients withdrew from the study and subsequent follow-up prior to surgery; 3 (4%) patients randomised to the control group versus 1 (1%) patient from the heated group.

In the control group, 60 (91%) patients had SSM, 2 (3%) had NSM; in the heated group, 63 (93%) had SSM and 5 (7%) had NSM. All incisions were periareloar in design. The median length of stay in the control group was 5 days, (interquartile range 3 to 5 days) and in the heated group was 5 days (interquartile range 4 to 5 days).

The outcomes of patients are summarised in Table 2. A total of 41 patients experienced post-operative necrosis; 23 in the control group versus 18 in the heated group. The proportion of patients experiencing necrosis in the control group was 35%, 95% CI [24 to 48%, *n* = 23/66]. In the heated group, the proportion of patients experience necrosis was 26%, 95% CI [17 to 39%, *n* = 18/68].

**Table 2:** Skin necrosis outcome at first occurrence and at 30–40-day follow-up

Necrosis outcome	*N* control/*N* heated	Control	Heated
Post-operative skin necrosis (*n*, %)	66*/68**		
No		43 (65%)	50 (74%)
Yes		23 (35%)	18 (26%)
Depth of necrosis at first occurrence (*n*, %)	62/64		
A—none		44 (71%)	50 (78%)
B—colour change		11 (18%)	9 (14%)
C—partial thickness		7 (11%)	4 (6%)
D—full thickness		0 (0%)	1 (2%)
Area of necrosis at first occurrence			
Total area if necrosis present (mm^2^)	18/14		
Median (IQR)		850.0 (100.0, 2700.0)	700.0 (400.0, 1300.0)
By SKIN score			
Area of colour change (mm^2^)	11/9		
Median (IQR)		300.0 (50.0, 1020.0)	700.0 (600.0, 1300.0)
Area of partial thickness (mm^2^)	7/4		
Median (IQR)		1500.0 (250.0, 3300.0)	730.0 (355.0, 2050.0)
Area of full thickness (mm^2^)	0/1		
Median (IQR)		–	–
Total area for all patients (including area = 0 mm^2^ for none)	61/64		
Median (IQR)		0.0 (0.0, 50.0)	0.0 (0.0, 0.0)
Maximum depth of necrosis over 30–40-day follow-up (*n*, %)	62/64		
A—none		44 (71%)	50 (78%)
B—colour change		10 (16%)	9 (14%)
C—partial thickness		6 (10%)	3 (5%)
D—full thickness		2 (3%)	2 (3%)
Maximum area of necrosis over 30–40 day follow-up			
Total area if necrosis present (mm^2^)	18/14		
Median (IQR)		850.0 (100.0, 2700.0)	700.0 (400.0, 1300.0)
By SKIN score			
Area of colour change (mm^2^)	11/9		
Median (IQR)		485.0 (50.0, 1020.0)	700.0 (600.0, 1300.0)
Area of partial thickness (mm^2^)	7/4		
Median (IQR)		1800.0 (250.0, 3300.0)	1000.0 (250.0, 3100.0)
Area of full thickness (mm^2^)	0/1		
Median (IQR)		(−)	(−)
Total area for all patients (including area = 0 mm^2^ for none)	61/64		
Median (IQR)		0.0 (0.0, 50.0)	0.0 (0.0, 0.0)
Necrosis resolved/fully healed within 30–40-day follow-up (*n*, %)	17/13		
No		8 (47%)	7 (54%)
Yes		9 (53%)	6 (46%)

*Necrosis outcome missing for *n* = 1 patient in control group who had SRM. The remaining *n* = 3 control patients without the necrosis outcome withdrew prior to surgery. **Necrosis outcome missing for *n* = 2 patients in heated group who had NSM or mastectomy only. The remaining *n* = 1 heated patient without the necrosis outcome withdrew prior to surgery. All measurements reported here by primary clinical assessor

In the control group, 4 of the 23 patients experiencing necrosis (17%, 95% CI [5 to 39%]) required surgical intervention for necrosis. In the heated group, 2 out of the 18 patients experiencing necrosis (11%, 95% CI [1 to 35%]) required surgical intervention for necrosis.

With regard to minimisation variables, those that experienced necrosis (*n* = 23 control; *n* = 18 heated) consisted of following:

- 3 (13%) smokers in controls compared to 2 (11%) in heated patients
- 1 (4%) diabetic in controls compared to none (0%) in heated patients
- 4 (17%) implant reconstructions in controls compared to 3 (17%) in the heated group
- 19 (83%) autologous reconstructions in controls compared to 15 (83%) in heated patients
- 3 (13%) in BRCA carriers in controls compared to 2 (11%) patients in the heated group. All these patients underwent NSM.

For full details on outcomes by minimisation variables please refer to Additional file 1: Table S5.

The individual ratings for the paired assessments of each comparison are displayed in Tables 3 and 4. For necrosis (yes/no) the kappa statistic indicated almost perfect agreement between the primary and secondary clinical assessor. For necrosis depth (SKIN score), the kappa statistic indicated substantial agreement between the primary and secondary clinical assessor. Bland and Altman plots (not presented) indicated a poor level of agreement between assessors for necrosis area.

**Table 3:** Individual ratings and Kappa statistic for the agreement of necrosis (yes/no) for primary clinical, secondary clinical and photographic 1 and 2 raters

Primary clinical	Secondary clinical		Kappa [95% CI]	Kappa interpretation
	No	Yes		
No	49	2		Almost perfect agreement
Yes	4	26	0.84 [0.62 to 1.00]	
Primary clinical	Photographic 1			
	No	Yes		
No	16	3		Substantial agreement
Yes	3	13	0.65 [0.32 to 0.99]	
Primary clinical	Photographic 2			
	No	Yes		
No	17	2		Moderate agreement
Yes	5	11	0.59 [0.26 to 0.92]	
Photographic 1	Photographic 2			
	No	Yes		
No	30	1		Substantial agreement
Yes	8	21	0.70 [0.45 to 0.94]

**Table 4:** Individual ratings and Kappa statistic for the agreement of necrosis depth (SKIN score) for primary clinical, secondary clinical and photographic 1 and 2 raters

Primary clinical	Secondary clinical				Kappa [95% CI]	Kappa interpretation
	None	Colour change	Partial thickness	Full thickness		
None	49	2	0	0		
Colour change	4	14	1	1		
Partial thickness	0	1	8	0		Substantial agreement
Full thickness	0	0	0	1	0.79 [0.58 to 0.99]	
Primary clinical	Photographic 1					
	None	Colour change	Partial thickness	Full thickness		
None	16	2	1	0		
Colour change	3	4	3	0		
Partial thickness	0	0	5	1		Moderate agreement
Full thickness	0	0	0	0	0.53 [0.26 to 0.80]	
Primary clinical	Photographic 2					
	None	Colour change	Partial thickness	Full thickness		
None	17	1	1	0		
Colour change	5	3	2	0		
Partial thickness	0	2	3	1		Fair agreement
Full thickness	0	0	0	0	0.40 [0.12 to 0.69]	
Photographic 1	Photographic 2					
	None	Colour change	Partial thickness	Full thickness		
None	30	0	1	0		
Colour change	6	6	2	0		
Partial thickness	2	4	4	0		Moderate agreement
Full thickness	0	0	3	2	0.50 [0.29 to 0.71]	

Table 5 displays the performance matrix used to aid decision making around the primary outcome for the definitive trial based on three pre-defined key criteria (agreement, sample size required, proportion with outcome measured). The agreement of necrosis outcome measure between assessors was more consistent with a ‘yes/no’ assessment compared to using the SKIN score or necrosis area (mm^2^). Based on the observed between group differences, power calculations indicate we would need 1096 patients to demonstrate a statistically significant difference using the yes/no assessment compared to 1556 patients using the SKIN score. An even higher sample size of 2866 would be required for necrosis area. The proportion of patients with an observed response was also highest for the yes/no assessment (95.0%) versus the SKIN score (89.4%) and necrosis area (88.7%).

**Table 5:** Performance matrix for necrosis outcomes

Criteria	Outcome measure		
	Necrosis yes/no	Depth (SKIN score)	Total necrosis area
Subjectivity of measurement (*κ* [95% CI] for the primary and secondary clinical assessor*)	0.84 [0.62 to 1.00]	0.79 [0.58 to 0.99]	0.57 [0.21 to 0.94]
Total sample size required to demonstrate statistically significant difference between treatment and control group (based on observed data)^†^	1096	1556	2866
Proportion of patients with observed response**	95.0%	89.4%	88.7%

*For total necrosis area, we include *κ* where area is assumed to be 0 mm^2^ when no necrosis present is recorded. ^†^For necrosis yes/no, the sample size was determined for a two-sample proportion test of 26% (heated) versus 35% (control). For total area, the sample size was computed using non-parametric methods for non-normally distributed continuous data. For necrosis, depth sample size calculation for ordered categorical data was performed using the observed proportions in each category (71, 18, 11 and 0% versus 78, 14, 6 and 2%). All sample size calculations use a 5% level of significance and 90% power. **For total necrosis area, area is assumed to be 0 mm^2^ where no necrosis present is recorded

### Compliance with heating protocol and outcome

Of the 67 patients with compliance data, 51 (76%) complied with the heating protocol and 16 (24%) did not. The reasons for non-compliance were varied and are presented within the Additional file 2: Table S6a and Additional file 3: Table S6b.

### Complications and safety outcome data

Table 6 summarises the safety data for the preHEAT trial. In total, there was one adverse reaction (AR), 30 serious adverse events (SAE; in 22 patients) and no serious adverse reactions (SAR). The one AR was a superficial burn caused to the breast due to incorrect use of the thermometer provided. This burn was managed non-operatively.

**Table 6:** Safety events’ data

Event	Control	Heated	Total
AR	0	1 (1)	1 (1)
SAE	10 (7)	20 (15)	30 (22)
SAR	0	0	0
Complication			
Mastectomy skin-flap necrosis	5	2	7
Infection	2	4	6
Haematoma	2	6	8
Re-do anastamosis	–	4	4
Flap failure	1	3	4
Burn	–	1	1

Number of patients shown in brackets as some patients had more than one safety event. *AR* adverse reaction related to heating protocol, *SAE* serious adverse event not related to heating protocol, *SAR* serious adverse reaction related to heating protocol. Note: *AE* adverse events (not serious and not related to heating protocol) were not recorded in the PREHEAT database

## Discussion

The rate of MSFN varies widely in the literature from between 5 and 30% [2, 12–19]. The rate of skin necrosis seen in this trial in both heated and non-heated groups (26 and 35% respectively) is in-line with other figures in the literature. In addition, there is no consistent nor accepted method for the measurement of necrosis that can be used to compare results between different surgical units. In order for us to determine if local heat preconditioning provides benefit to patients, we needed to explore the various methods for MSFN outcome measurement that would inform the design of a robust protocol for a multi-centre definitive trial in the future. It would also help us estimate the number of patients we need to power the study.

preHEAT was designed as a feasibility trial to develop such a protocol. This trial shows there is near perfect agreement between assessors in clinic using the yes/no assessment. The performance matrix showed that skin necrosis of any depth categorised as ‘yes/no’ required substantially less participants to power a definitive trial, agreement of assessment was higher and the outcome had less missing data compared to the SKIN score and measuring necrosis area. Following extensive discussion of these results in a Trial Steering Committee meeting, we therefore suggest that the most suitable assessment of MSFN as the primary outcome is the binary measurement of yes/no necrosis and the need for surgical intervention as a secondary outcome measure in a definitive trial.

Blinding in surgical trials has always been a challenge [20]. We were acutely aware that the assessment process must be as effective as possible in order to minimise assessor bias. We opted for a simple sealed envelope system to record outcomes that was easy to administer, and we concentrated on the training of all assessors in its use. We found that this system worked well and will use it for the multicentre trial and would recommend such a system for other groups wishing to ensure blinding during assessment.

There was good compliance with the heating protocol overall, and we attribute this to both the clear instructions given to patients and to the simplicity of local heat preconditioning itself. Some patients who did not comply cited reasons of not being able to use the thermometer correctly, but this was only the case for 4 patients. We believe that the preHEAT has demonstrated that the method of heat preconditioning using hot water bottles and thermometers is acceptable to patients and has a high level of compliance that would be appropriate for use in a larger definitive trial (Additional file 4).

The rate of MSFN in the heated group was lower compared to controls, and we observed that the rate of return to theatre for those patients that developed necrosis was lower in the heated group. This suggests that there is a putative benefit in heat preconditioning and the reduction in the number of patients requiring surgical intervention. This could have substantial consequences for patients and the NHS as a whole in the form of reducing the need for return to theatre and subsequent costs.

Regarding the safety of local heat preconditioning using hot water bottles, one patient unfortunately experienced a superficial burn. Further investigation of this case indicated the thermometer was damaged by being immersed in boiling water and therefore was probably not able to read the temperature of the water correctly. We had stipulated explicitly in the Patient Information Sheet that if there were any problems they can contact one of the research team. This was not done. The burn was very small and on an area of the skin excised routinely during the mastectomy. This single unfortunate event highlighted the importance of following the heating protocol closely and notifying the research team of faulty equipment. However, this trial still demonstrates that this method of preconditioning is safe overall.

Closer examination of the surgical complications experienced during this trial reveals the most striking observation which is a higher post-operative haematoma rate in the heated group compared to controls. This was scrutinised by the Trial Steering Committee mid-way through the trial and concluded to be surgery-related. A literature search indicated that the rate of haematoma requiring surgical intervention was in line with other studies and therefore is not likely to be increased by the heating process [14, 21, 22]. No other complications, SAEs, SARs or ARs were attributable to the heating procedure; therefore, we can conclude that this is a safe intervention.

### Limitations of the study

This feasibility study was not powered to detect a significant difference in MSFN and heat preconditioning. However, the study has demonstrated a positive signal that shows a possible beneficial effect of heat preconditioning on MSFN rates. The future plans for this trial are to expand to a definitive multicentre RCT through further grant applications such as the Health Technology Assessment funding stream with the NIHR.

## Conclusion

The results from this trial inform us that the binary outcome of MSFN “yes/no” is a suitable and reliable primary outcome measure of necrosis and was superior to the SKIN Score or necrosis area. The trial protocol was found to be feasible, and there is a positive signal towards a beneficial effect of local heat preconditioning on wound healing outcome that warrants further investigation in a larger, multicentre, definitive randomised controlled trial. We have subsequently started funding applications to the NIHR.

## Additional files

Additional file 1: **Table S5.** Minimisation variable outcomes. Data showing the outcomes for patients with regard to minimisation variables. (DOCX 57 kb)

Additional file 2: **Table S6a.** Reasons for non-compliance with heating protocol. (DOCX 47 kb)

Additional file 3: **Table S6b.** “Other” reasons for non-compliance with heating protocol. (DOCX 49 kb)

Additional file 4: Compliance document given to patients for the heating protocol. (PDF 179 kb)

## References

[1] [NG101], N. I. C. E. G. Early and locally advanced breast cancer: diagnosis and management. *NICE Guideline* [NG101], (2018).

[2] Jeevan R (2014). Findings of a national comparative audit of mastectomy and breast reconstruction surgery in England. J Plast Reconstr Aesthet Surg.

[3] Contaldo C (2007). The influence of local and systemic preconditioning on oxygenation, metabolism and survival in critically ischaemic skin flaps in pigs. J Plast Reconstr Aesthet Surg.

[4] Harder Y (2004). Improved skin flap survival after local heat preconditioning in pigs. J Surg Res.

[5] Harder Y (2005). Heat shock preconditioning reduces ischemic tissue necrosis by heat shock protein (HSP)-32-mediated improvement of the microcirculation rather than induction of ischemic tolerance. Ann Surg.

[6] Harder Y (2008). An old dream revitalised: preconditioning strategies to protect surgical flaps from critical ischaemia and ischaemia-reperfusion injury. J Plast Reconstr Aesthet Surg.

[7] Mehta S, Rolph R, Cornelius V, Harder Y, Farhadi J (2013). Local heat preconditioning in skin sparing mastectomy: a pilot study. J Plast Reconstr Aesthet Surg.

[8] Cro S, Mehta S, Farhadi J, Coomber B, Cornelius V (2018). Measuring skin necrosis in a randomised controlled feasibility trial of heat preconditioning on wound healing after reconstructive breast surgery: study protocol and statistical analysis plan for the PREHEAT trial. Pilot Feasibility Stud.

[9] Lemaine V (2015). Introducing the SKIN score: a validated scoring system to assess severity of mastectomy skin flap necrosis. Ann Surg Oncol.

[10] Fleiss JL (1971). Measuring nominal scale agreement among many raters. Psychol Bull.

[11] Campbell MJ, Julious SA, Altman DG (1995). Estimating sample sizes for binary, ordered categorical, and continuous outcomes in two group comparisons. BMJ.

[12] Antony AK (2009). Salvage of tissue expander in the setting of mastectomy flap necrosis: a 13-year experience using timed excision with continued expansion. Plast Reconstr Surg.

[13] Garwood ER (2009). Total skin-sparing mastectomy: complications and local recurrence rates in 2 cohorts of patients. Ann Surg.

[14] Meretoja TJ (2007). Late results of skin-sparing mastectomy followed by immediate breast reconstruction. Br J Surg.

[15] Munhoz AM (2007). Immediate skin-sparing mastectomy reconstruction with deep inferior epigastric perforator (DIEP) flap. Technical aspects and outcome. Breast J.

[16] Patel KM, Hill LM, Gatti ME, Nahabedian MY (2012). Management of massive mastectomy skin flap necrosis following autologous breast reconstruction. Ann Plast Surg.

[17] Matsen CB (2016). Skin flap necrosis after mastectomy with reconstruction: a prospective study. Ann Surg Oncol.

[18] Robertson SA, Jeevaratnam JA, Agrawal A, Cutress RI (2017). Mastectomy skin flap necrosis: challenges and solutions. Breast Cancer (Dove Med Press).

[19] Wang F (2014). Total skin-sparing mastectomy and immediate breast reconstruction: an evolution of technique and assessment of outcomes. Ann Surg Oncol.

[20] Cook JA (2009). The challenges faced in the design, conduct and analysis of surgical randomised controlled trials. Trials.

[21] Halle M, Docherty Skogh AC, Friberg A, Edsander-Nord Å (2016). Breast free flap complications related to haematoma formation - do the risks of multiple antithrombotics outweigh the benefits today. J Plast Surg Hand Surg.

[22] Sotheran WJ, Rainsbury RM (2004). Skin-sparing mastectomy in the UK--a review of current practice. Ann R Coll Surg Engl.

