# Supplementary material for: A randomised controlled feasibility trial to evaluate local heat preconditioning on wound healing after reconstructive breast surgery: the preHEAT trial
Source: Pilot Feasibility Stud. 2019 Jan 11;5:5. doi: 10.1186/s40814-019-0392-y (PMC6329155; doi:10.1186/s40814-019-0392-y)
Supplement: Supplementary file 1 — Table S5. Minimisation variable outcomes. Data showing the outcomes for patients with regard to minimisation variables. (DOCX 57 kb) [file 40814_2019_392_MOESM1_ESM.docx]

Table 5 – Minimisation variables and outcomes

| **Minimisation variable** | **Control** | | **Heated** | |
| --- | --- | --- | --- | --- |
|  | **Necrosis (N=23)**  **N (%)** | **None**  **(N=43)**  **N (%)** | **Necrosis**  **(N=18)**  **N (%)** | **None**  **(N=50)**  **N (%)** |
| Smoking |  |  |  |  |
| Yes | 3 (13%) | 4 (9%) | 2 (3%) | 7 (14%) |
| No | 20 (87%) | 39 (91%) | 16 (20%) | 43 (86%) |
| Diabetic |  |  |  |  |
| Yes | 1 (4%) | 0 (0%) | 0 (1%) | 4 (8%) |
| No | 22 (96%) | 43 (100%) | 18 (22%) | 46 (92%) |
| Type of reconstruction |  |  |  |  |
| Implant | 4 (17%) | 9 (21%) | 3 (4%) | 10 (20%) |
| Autologous | 19 (83%) | 34 (79%) | 15 (19%) | 40 (80%) |
| BRCA carrier status |  |  |  |  |
| Yes | 3 (13%) | 10 (23%) | 2 (3%) | 9 (18%) |
| No | 20 (87%) | 33 (77%) | 16 (20%) | 41 (82%) |

Note: percentages are rounded so might not always sum to 100%.
