# Supplementary material for: A randomised controlled feasibility trial to evaluate local heat preconditioning on wound healing after reconstructive breast surgery: the preHEAT trial
Source: Pilot Feasibility Stud. 2019 Jan 11;5:5. doi: 10.1186/s40814-019-0392-y (PMC6329155; doi:10.1186/s40814-019-0392-y)
Supplement: Supplementary file 2 — Table S6a. Reasons for non-compliance with heating protocol. (DOCX 47 kb) [file 40814_2019_392_MOESM2_ESM.docx]

Table 6a Reasons for non-compliance with heating protocol

| **Reason for non-compliance** | **N** | **%** |
| --- | --- | --- |
| Lost the protocol sheet | 0 | 0% |
| Forgot to record the time and temperature | 1 | 6% |
| Heated less than 3 times | 1 | 6% |
| Did not have time to follow protocol before surgery | 2 | 12% |
| Heated wrong breast | 1 | 6% |
| Heated both breasts | 0 | 0% |
| Unable to use thermometer | 4 | 25% |
| Decided not to participate | 3 | 19% |
| Forgot to do heating protocol | 1 | 6% |
| Unable to record time accurately | 0 | 0% |
| Other | 10 | 63% |

Note: Some patients gave more than one of the listed reason for non-compliance.
