# Supplementary material for: A randomised controlled feasibility trial to evaluate local heat preconditioning on wound healing after reconstructive breast surgery: the preHEAT trial
Source: Pilot Feasibility Stud. 2019 Jan 11;5:5. doi: 10.1186/s40814-019-0392-y (PMC6329155; doi:10.1186/s40814-019-0392-y)
Supplement: Supplementary file 3 — Table S6b. “Other” reasons for non-compliance with heating protocol. (DOCX 49 kb) [file 40814_2019_392_MOESM3_ESM.docx]

Table 6b Other reasons for non-compliance with heating protocol

| **Reason for non-compliance** | **N** | **%** |
| --- | --- | --- |
| Heated twice then thermometer didn’t work | 1 | 6% |
| Heated x3 with thermometer damaged by being submerged in hot water | 1 | 6% |
| Listed for different surgery - BCS | 1 | 6% |
| Stayed at friends house evening before surgery | 1 | 6% |
| Skin reaction | 1 | 6% |
| Stayed in hotel evening before surgery | 1 | 6% |
| Surgery changed to mastectomy only | 1 | 6% |
| Surgery changed to NSM | 1 | 6% |
| Unable to ascertain how patient heated as she cannot recall | 1 | 6% |
| Upset as argued with partner, so decided not to participate | 1 | 6% |
