# Supplementary material for: A randomised controlled feasibility trial to evaluate local heat preconditioning on wound healing after reconstructive breast surgery: the preHEAT trial
Source: Pilot Feasibility Stud. 2019 Jan 11;5:5. doi: 10.1186/s40814-019-0392-y (PMC6329155; doi:10.1186/s40814-019-0392-y)
Supplement: Supplementary file 4 — Compliance document given to patients for the heating protocol. (PDF 179 kb) [file 40814_2019_392_MOESM4_ESM.pdf]

preHEAT

Patient ID:

Date of birth:

# PREHEAT Heating Procedure

*Things to remember:*

1. If you are having **only one breast** operated on, then heat **only that one**.
2. If you are having **both breasts** operated on, then heat only your **RIGHT** breast.
3. Heat the breast around **12 hours** before surgery, i.e. at around 7pm the night before.
4. The heating procedure takes about **2 hrs 45 mins**, please allow enough time to complete the entire procedure.
5. Get someone to help you if you need to.
6. **Lie down** and **relax** during the heating procedure.
7. Please write down the **temperature** you heated the water to, the **time** the hot water bottle was placed on and off the breast and **which breast** you heated in the spaces provided on the next page.
8. Any problems, call our clinical research nurse **Ms Billie Coomber** at **07825 016051**.

**What you need to complete the procedure**

- A saucepan with water
- The thermometer we have given you
- The water bottle we have given you
- A tea towel
- A pen
- A watch / timer
- This form

# INSTRUCTIONS

## PART ONE

1. Place the thermometer screen away from the hob and make sure the screen shows ‘**OUT**’ and the temperature in degree Celsius (°**C**). If not, press on the **IN/OUT** once or the **°C/°F** button once to switch to the correct setting.
2. Extend the thermometer probe into the water in the saucepan and make sure the probe does not touch the saucepan.
3. Heat water to **43°C**.
4. Record the water temperature overleaf.
5. **Half** fill the bottle slowly.
6. Cover the bottle with a tea towel.
7. Lie down and place on the naked breast with the centre of the bottle over your nipple for **30 minutes**.
8. Write down overleaf the **time** the hot water bottle was placed **on** and **off** the breast and which breast you heated.

## BREAK

9. Break for **30 minutes** and allow your breast to cool on its own.

## PART TWO

10. Tip out the water from the water bottle.
11. **Repeat Part One**, steps 2 to 7 with freshly heated water.
12. Record the temperature and time overleaf.

## BREAK

13. Break for **30 minutes** and allow your breast to cool on its own.

## PART THREE

14. Tip out the water from the water bottle.
15. **Repeat Part One**, steps 2 to 7 with freshly heated water
16. Record the temperature and time overleaf.

# PLEASE COMPLETE THIS PAGE

**Which** breast did you heat?

**RIGHT / LEFT** (circle)

Today's date:

/ /

## PART ONE

Temperature of water: °C

Time hot water bottle was placed on breast and was taken off:

**on:** : **PM** **off:** : **PM**

Any comments / difficulties?

## PART TWO

Temperature of water: °C

Time hot water bottle was placed on breast and was taken off:

**on:** : **PM** **off:** : **PM**

Any comments / difficulties?

## PART THREE

Temperature of water: °C

Time hot water bottle was placed on breast and was taken off:

**on:** : **PM** **off:** : **PM**

Any comments / difficulties?

Please bring this **completed form**, the **water bottle** and the **thermometer** with you when you come to the hospital for your surgery. A member of the research team will collect these from you during your stay at the hospital.

Thank you very much for your participation!
